# Dating of groundwater using uranium isotopes disequilibrium in Siwa Oasis, Western Desert, Egypt

**DOI:** 10.1038/s41598-023-39333-w

**Published:** 2023-07-31

**Authors:** Noha Imam

**Affiliations:** National Institute of Oceanography and Fisheries, 101 Kaser El Aini Street, Cairo, 11516 Egypt

**Keywords:** Environmental sciences, Physics

## Abstract

Data on the recent migratory history of radionuclides as well as geochemical circumstances can be obtained from the disequilibrium of the uranium series, which is often brought on by groundwater flow and host rock. Groundwater from the Siwa Oasis is a vital source of water for many uses, and it is distributed widely throughout the Western Desert. Groundwater in Siwa Oasis was dated using measurements of uranium in the water. In water samples that exhibited disequilibrium behavior, the activity concentrations of radionuclides from the ^238^U, ^235^U and ^232^Th series were measured. Therefore we conclude that the measured waters are rich in the ^234^U and ^230^Th. The secular equilibrium between ^234^U and ^230^Th indicates that colloidal transport could be the mechanism for the mobility of ^230^Th in groundwater. Higher ^230^Th levels in the samples show that the aquifer is deep and may have a large amount of thorium-bearing minerals. The lake and groundwater estimated ages showed that the time of uranium migration happened between 60 and 130 ka ago. This aquifer is rich in mineral deposits, as evidenced by the extraordinarily high content of radionuclides. The ^230^Th/^232^Th activity ratio of the samples, indicating pure carbonate minerals, ranged from 12.58 to 20.86.

## Introduction

Siwa is the last natural oasis surviving in Egypt, and it depends primarily on groundwater resources and recycled drainage water. Groundwater has always been a topic of serious study due to its importance as a national resource and as part of the overall water cycle^1^, especially in the arid regions of recent decades as human population increases in desert areas^2^. There are about 200 natural springs and shallow and deep wells in the Siwa Oasis, but only about 80 of them are used for irrigation, drinking (Roman Eyes) and medical treatment (Sulphur Springs), with Ain Cleopatra being the best known^3^. The shallow limestone aquifer, known for its highly saline water, is the source of more than 1200 wells. However, fresh water is found in the deep Nubian sandstone aquifer^4^. Our understanding of the systems and age of groundwater has improved with the development of methods based on stable and radioactive isotope measurements^2^. The age of groundwater from the Nubian Sandstone aquifer has been estimated to be between 10,000 and 33,000 years^5^, between 25,000 and 40,000 years^6^ and between 200,000 and one million years^7^ using various techniques.

All groundwater contains natural radioactivity (U and Th series), and these radionuclides have a very long half-life, which makes it possible to date events that occurred in different time periods. It is often expected that the radionuclides in each decay series are in secular equilibrium with their daughters. However, these radionuclides exhibit considerable relative fractionation in the groundwater surrounding them, reflecting different behavior both in their release to the water and in their interaction with the aquifer rock^1^.

Therefore, groundwater disequilibrium provides data for radionuclide migration studies, including: (1) its recent history, (2) factors affecting radionuclide migration, and (3) the geochemical properties of the water–rock systems in which it occurred^8,9^. A great deal of differentiation among the U-series nuclides arises from processes during water–rock interactions, reflecting their different chemical properties when they enter the water phase^10,11^. Natural waters usually suffer from fractionation of the uranium series (U) into parent-daughter pairs, ^234^U and ^238^U, and the disequilibrium between these is often used as an indicator of groundwater movement^12^.

### Theory of U-series dating

U-series dating is a radiometric dating method often used to estimate the date of an "event" that occurred during an elemental fractionation process corresponding to weathering processes^13^. This approach is based on the decay of U-238, which has a half-life of 4.469 × 10^9^ a, to reach the stable Pb-206 via transition daughters such as ^234^U and ^230^Th, which have half-lives of 245,000 and 75,400 a, respectively. In this decay series, a disequilibrium between U and Th occurs when U and Th differ due to a different geographical or climatic event. The system reaches near-secular equilibrium only after seven times the half-life of Th-230 (500 Ka), after this radioactive disequilibrium has occurred. The radioactive disequilibrium between nuclides in the decay series is a marker for earlier fractionation processes, which are typically associated with the increase or decrease of highly migrating isotopes^14^.

This process is described via the Bateman Eq. (1)^15–17^.

1 $$ \frac{{{}^{230}Th}}{{{}^{234}U}} = \left[ {\left( {\frac{1}{{{}^{234}U/{}^{238}U}}} \right) \times \left( {1 - e^{{ - \left( {\lambda_{230} *T} \right)}} } \right)} \right] + \left\{ {\left( {1 - \frac{{{}^{238}U}}{{{}^{234}U}}} \right) \times \left( {\frac{{\lambda_{230} }}{{\lambda_{230} - \lambda_{234} }}} \right) \times \left( {1 - e^{{ - \left( {\lambda_{230} - \lambda_{234} } \right)*T}} } \right)} \right\} $$

Here T is the age of the sample, the activity ratios are ^238^U/^234^U and ^230^Th/^234^U, and λ_230_ and λ_234_ are the corresponding decay constants of ^230^Th and ^234^U.

The age determined using the above equation is valid if the following assumptions are fulfilled (1) the U/Th isotope system has remained closed since the U/Th fractionation event; (2) the U/Th fractionation "event" is a rapid process compared to elapsed time; and (3) the U/Th fractionation or dated material does not contain an initial inherited ^230^Th at the time of formation or this initial ^230^Th is small^18^. In a particular scenario of U/Th dating, one phase is assumed to contain all initial thorium (detrital fraction), while the other phase contains only thorium obtained by radioactive decay (authigenic fraction)^19^. The implications of detrital contamination could be mitigated by determining the activity of ^232^Th, which occurs only in the detrital fraction but plays no role in the U-238 decay series^20^. ^234^U had already decayed to ^230^Th* (radiogenic) during ageing. The activity concentration of ^230^Th* was determined from the total activity concentration of ^230^Th subtraction Th (detrital) using Eq. (2). The uncorrected ^230^Th/U age calculated from the difference between the ^230^Th total activity concentration and the detrital corrected ^230^Th*/U age determined from (^230^Th*).

2 $$ \left[ {{}^{230}Th} \right]_{Total} = \left[ {{}^{230}Th} \right]^{*} + \left\{ {\left( {\frac{{{}^{230}Th}}{{{}^{232}Th}}} \right) \times^{232} Th} \right\} = \left[ {{}^{230}Th} \right]^{*} + \left\{ {f \times^{232} Th} \right\} $$

Consequently, for a more thorough understanding of the behavior of uranium-series radionuclides in the groundwater system of Siwa Oasis. This work aims to calculate the uranium age dating method for water by using U-series disequilibria and the activity concentration of U-series isotopes in the water of the western desert of Siwa Oasis.

## Materials and methods

### Geological setting and sampling

**Figure 1:**
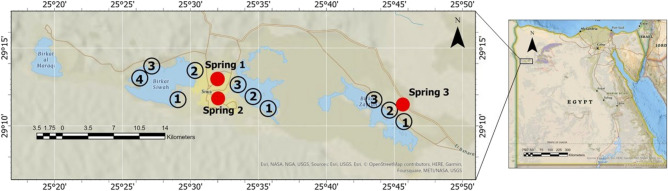
Map showing sampling sites using ArcGIS Pro 3.1^®^ software by Esri. ArcGIS^®^ and ArcMapTM are the intellectual property of Esri (Copyright © Esri. All right reserved^24^).

The Siwa Oasis is a large depression in the northwest of the Western Desert of Egypt at latitudes 29° 05′ 00″ N and 29° 25′ 00″ N and longitudes 25° 05′ 00″ and 26° 06′ 00″ E^21^. There are two major aquifers: the shallow aquifer (the upper Siwa) and the deepest aquifer (Nubian sandstone). Springs are located in the shallow Middle Miocene carbonates, which are completely isolated from the Nubian Sandstone (the deep aquifer)^22^. The Mesozoic and Palaeozoic periods are indicated by the thickness of the Nubian aquifer of 2600 m. Geologically, the Siwa Oasis carbonate aquifer system is primarily composed of hard limestone, with limestone occurring in some areas^23^. In January 2018, samples were collected from three freshwater springs and three hypersaline lakes in Siwa Oasis (Fig. 1). The Siwa Lakes (hypersaline lakes) are watersheds that collect water from cultivated areas and groundwater from wells and springs. More than three samples were collected at each site. The water samples were collected and filled into a plastic bottle (2L). All samples were kept in the icebox until returned to the laboratory. The maps were created using ArcGIS Pro 3.1^®^ software by Esri. ArcGIS^®^ and ArcMapTM are the intellectual property of Esri and are used here in under license. Copyright © Esri. All right reserved. For more information about Esri^®^ software, please visit^24^

### Analytical techniques

The HPGe detector is a non-destructive method that has a relative efficiency of roughly 60% in comparison to the 3 × 3 NaI (Tl) crystal efficiency with a resolution (FWHM) of 1.90 keV, and peak/Compton ratio of 69.9:1 at the 1.33 MeV gamma transition of ^60^Co. he detector was shielded from background radiation with a 10-cm-thick lead liner lined internally with a 2-mm-thick copper foil to absorb X-rays. The certified standard sources of IAEA, RGU -I, RGTh-I, and RGK-I provided by^25^, were used for the energy and efficiency calibration of the system, which covers the energy range of 46.53–3000 keV. The Gamma spectrum were recorded and analyzed using MAESTRO software from ORTEC, and the radioactive concentration was calculated manually using a spreadsheet (Microsoft Excel). To prevent contamination, marinelli beakers were cleaned, rinsed with diluted H_2_SO_4_, and dried before being filled with known volumes of water. The marinelli beakers have been firmly sealed within 4 weeks. In order to prevent radon leakage and ensure that the Uranium series and their progeny were in a condition of secular equilibrium. The activity concentration of radionuclides in water represented as Bq/l and the accumulation time for measuring was within 48–72 h. The activity concentration of ^238^U was calculated via ^234m^Pa, whose the activity was determined from 1001 keV photo peaks. Gamma-ray peaks for ^235^U at 143.8, 163.4, 185.7, and 205.3 keV were used to estimate its activity^26,27^.

The activity concentration of ^234^U was determined via 53.2 keV (after subtraction) and 120.9 keV (after the subtraction)^28^. The 67.7 keV peak was used to calculate the activity of ^230^Th^29^. Gamma energies of 338.4 keV and 911.2 keV for ^228^Ac, 583 keV and 2614.4 keV for ^208^Tl, and 727.3 keV for ^212^Bi were used to measure the activity concentration of ^232^Th series. The concentrations of radionuclides were calculated using the equation of^30^. IAEA- reference materials (IAEA-443, IAEA-446, and IAEA-410) were used to confirm the accuracy and validity of the analytical data reported in this study, as showed in Table 1. The measuring system’s lowest limits of detection (LLDs), which must be calculated in order to determine a minimum detection level for each sample's radionuclide content using an analytical method, were obtained by^31^. The obtained LLD values of water are 0.017, 0.01, and 0.005 Bq/l for ^238^U, ^232^Th, and ^235^U, respectively.

**Table 1:** The quality control of the analytical data (Bq/kg).

Sample code	This analysis measured values (Bq/kg)					Certified reference values (Bq/kg)				
	^238^U	^234^U	^235^U	^230^Th	^232^Th	^238^U	^234^U	^235^U	^230^Th	^232^Th
IAEA-446	9.89 ± 0.5	11.1 ± 0.98	0.46 ± 0.3	0.39 ± 0.3	0.42	9.34	10.5	0.44	0.36	0.38
IAEA-443	0.04 ± 0.002	0.05 ± 0.009	0.00188 ± 0.0002	–	0.22	0.039 ± 0.002	0.044 ± 0.002	0.00185 ± 0.0001	–	0.19
IAEA-410	10.4 ± 0.93	10.2 ± 1.3	–	–	9.3 ± 0.62	10.1 ± 0.8	10.0 ± 1	–	–	8.70 ± 0.91

## Results and discussion

### Environmental parameters

Summary of the physicochemical parameters of the water samples of the study area (Table 2). For hypersaline lakes, the temperature of water samples ranged from 16.5 to 19.5 °C, while for wells, it ranged from 31.2 to 53.2 °C. Groundwater flowing into the well from a hydraulically conductive fracture or aquifer will be warmer than the temperature at the gauge because temperature increases with aquifer depth, and vice versa^32^. The pH of the water samples varied from 7.42 to 7.92, with an average value of 7.68, indicating a neutral to slightly alkaline pH. The acceptable pH range for the groundwater samples of the well was within the range of (6.5–8.5)^33^. The total dissolved solid (TDS) measurement ranged from 0.8 to 198.61 g/l, with an average value 181.21 g/l for hypersaline lakes and 1.3 g/l for wells. The US Geological Survey recommends categorizing groundwater based on TDS as fresh (1000 mg/L), slightly saline (1000–3000 mg/L), moderately saline (300–10,000 mg/L), and extremely saline (10,000–35,001 mg/L)^34^. The classification of groundwater indicated that the groundwater of this study slightly salinized.

**Table 2:** The values of pH, temperature and total dissolved solid (TDS) of water samples.

Samples	Temp. (ºC)	pH	TDS (g/l)
Zietoun Lake (1)	19.5	7.61	198.61
Zietoun Lake (2)	17.5	7.54	187.50
Zietoun Lake (3)	18.7	7.68	193.2
Aghormi Lake (1)	19.1	7.92	178.47
Aghormi Lake (2)	18.7	7.74	183.4
Aghormi Lake (3)	18.6	7.67	176.3
Siwa Lake (1)	19.4	7.81	165.8
Siwa Lake (2)	16.5	7.76	173.4
Siwa Lake (3)	17.5	7.67	176.5
Siwa Lake (4)	18.9	7.83	178.9
Spring (1)	31.2	7.62	1.4
Spring (2)	42.6	7.54	0.8
Spring (3)	53.2	7.42	1.8

### Activity concentration of radionuclides

**Figure 2:**
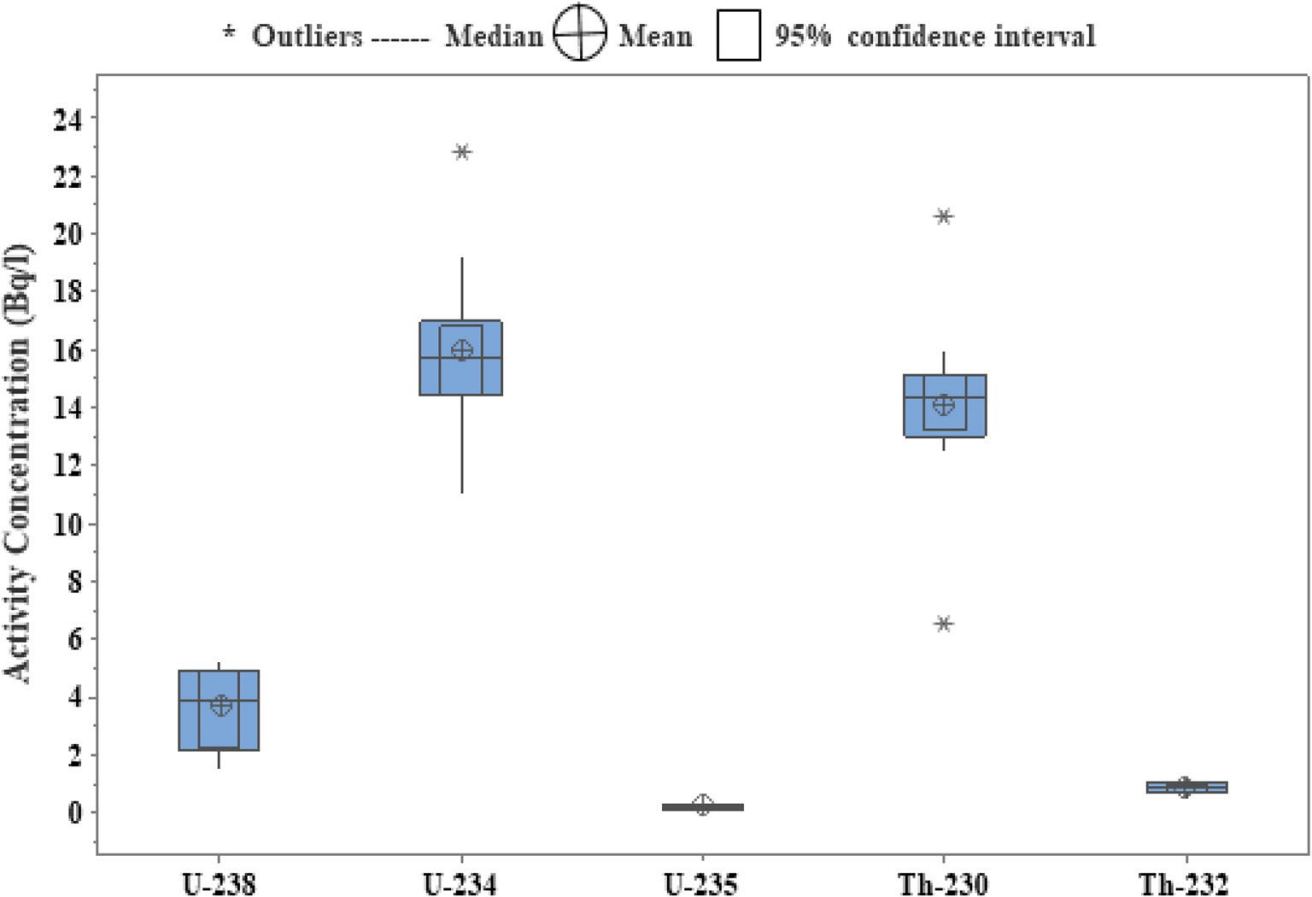
Boxplot of ^238^U,^234^U,^235^U,^230^Th and ^232^Th in water samples of Siwa Oasis.

The activity concentrations of ^238^U, ^234^U, ^235^U, ^230^Th, and ^232^Th in water samples expressed as Bq l^−1^ were shown in Table 3 and Fig. 2. The radionulcides concentration of ^238^U,^234^U and ^235^U ranged from 1.9 ± 0.49 Bq l^−1^ at Siwa (1) to 5.24 ± 0.90 Bq l^−1^ at Zietoun (1), from 11.01 ± 3.67 Bq l^−1^ at Spring (3) to 19.23 ± 6.41 Bq l^−1^ at Siwa (4) with an outlier 22.92 ± 7.64 Bq l^−1^ at Spring (1), and from 0.07 ± 0.05 Bq l^−1^ at Siwa (4) to 0.24 ± 0.14 Bq l^−1^ at Zietoun (1), respectively. While, the ranges of activity concentration of Thorium in the water samples were nearly narrow ranges from 12.45 to15.9 Bq l^−1^ for ^230^Th with an outlier 6.5 ± 2.17 Bq l^−1^ at Spring (3) and 20.67 ± 6.89 Bq l^−1^ at Spring (1). Also, the activity concentration of ^232^Th ranged from 0.4 ± 0.19 Bq l^−1^ at Spring (3) to 1.2 ± 0.37 Bq l^−1^ at Siwa (2). According to these results, the activity concentration of radionuclides of U-series in all water samples indicated that ^234^U =  > ^230^Th > ^238^U due to the variations in groundwater geochemical conditions brought on by water–rock interaction. The differences in the chemical and physical properties of the individual radionuclides, which affect their ability to dilute and move in water, are the main reason for the disequilibrium in the U-238 series^35^. The solubility, adsorption, desorption and precipitation of a nuclide are influenced by its chemical composition. The flow of water along a particular pathway is affected over time by fractionation due to the difference in solubility of the oxidized U isotopes compared to Th and relative to ^234^U and ^238^U, respectively^36^. The crystal lattice of rocks exposed to water interaction is altered by recoil displacement or oxidation, preferential mobilisation of ^234^U relative to ^238^U, adding or removal of U comparative to Th, and chemical fractionation of ^234^Th^37^. In these samples, there is an enrichment in ^234^U compared to ^238^U in groundwater may due to the two processes: (a) the direct release of ^234^Th from an aquifer mineral into solution followed by in situ decay to Uranium; or (b) the growth of a recoil damaged lattice site for ^234^Th (and thus for ^234^U) in aquifer mineral, which would be highly sensitive to preferential leaching^38^.

**Table 3:** The activity concentration of radionuclides in water samples (Bq/l).

Sample no	^238^U	^234^U	^235^U	^230^Th	^232^Th
Zietoun Lake (1)	5.24 ± 0.90	16.24 ± 5.41	0.24 ± 0.14	14.35 ± 4.78	0.7 ± 0.27
Zietoun Lake (2)	5.1 ± 0.89	14.22 ± 4.74	0.24 ± 0.14	13.56 ± 4.52	0.65 ± 0.26
Zietoun Lake (3)	4.95 ± 0.87	16.55 ± 5.52	0.23 ± 0.14	14.2 ± 4.73	0.75 ± 0.28
Aghormi Lake (1)	4.02 ± 0.77	14.65 ± 4.88	0.18 ± 0.12	15.2 ± 5	0.8 ± 0.29
Aghormi Lake (2)	3.89 ± 0.75	15.45 ± 5.15	0.19 ± 0.12	14.89 ± 4.96	0.9 ± 0.31
Aghormi Lake (3)	3.5 ± 0.71	15.89 ± 5.30	0.16 ± 0.11	12.5 ± 4.17	0.7 ± 0.27
Siwa Lake (1)	1.9 ± 0.49	14.5 ± 4.83	0.09 ± 0.05	13.5 ± 4.50	0.9 ± 0.31
Siwa Lake (2)	2.3 ± 0.55	17.4 ± 5.80	0.11 ± 0.05	15.1 ± 5.03	1.2 ± 0.37
Siwa Lake (3)	1.8 ± 0.47	15.7 ± 5.1	0.09 ± 0.05	14.5 ± 4.83	1.1 ± 0.35
Siwa Lake (4)	1.5 ± 0.43	19.23 ± 6.41	0.07 ± 0.05	15.9 ± 5.30	0.8 ± 0.29
Spring (1)	4.74 ± 0.85	22.92 ± 7.64	0.23 ± 0.14	20.67 ± 6.89	1.1 ± 0.35
Spring (2)	3.32 ± 0.68	14.37 ± 4.79	0.15 ± 0.09	12.45 ± 4.15	0.64 ± 0.26
Spring (3)	4.89 ± 0.86	11.01 ± 3.67	0.22 ± 0.13	6.5 ± 2.17	0.4 ± 0.19

Additionally, contrary to the expectations, it is evident that the activity concentration of ^230^Th was almost similar to the ^234^U activity concentration. Thorium content in water is supposed to be relatively low due to low solubility, but there are a few studies that indicated the solubility of thorium in groundwater, such as the basaltic Snake River aquifer^39^, saline groundwaters from Missouri carbonates and sandstones^38^, and unconsolidated sandy aquifers^40^. According to EDTA data, Th solubility can be increased by complexing with organic ligands^36^ and humic or carbonate materials^38^. Moreover, the high concentrations of ^230^Th in this study are in agreement with the analysis of the groundwater of the Bahariya and Farafra oases in Egypt's Western Desert^41^. Whereas; The Th concentration in the waters of the Bahariya Oasis is more than ten times higher than the U concentration and The Farafra Oasis have an average of Th concentration that is a lower than the equivalent U levels, but still quit high compared to the worldwide averages. This extraordinary level of Thorium indicated that there are Th-rich minerals in the lowest depths of the aquifer, but not necessarily ore deposits.

This implies that the deep aquifer rocks are the source of the ^230^Th, and chemical processes are coordinating the mobility of ^230^Th rather than recoil processes. On the same context, the only way of released ^232^Th from the aquifer minerals by weathering, and the existence of ^232^Th in aquifers although a high removal processes from groundwater indicates that continual release happens^1^. The presence of Th-bearing minerals in the sandstone aquifer is confirmed by the extremely high values for the ^232^Th-series in the water^42^, and the comparatively high ^232^Th levels in some of the groundwaters must be the consequence of more recent desorption processes (within the past 30 years)^1^. The ranges of U and Th concentrations in these samples are significantly higher compared to those typical for groundwater^43^, and Thorium may be associated with colloids rather than in realistic media, as suggested by [^44^] for the aquifer waters of the Western Desert of Egypt. The Uranium and Thorium can be considered to have come from the crystalline host rocks, just like the other dissolved ions^45^.

**Figure 3:**
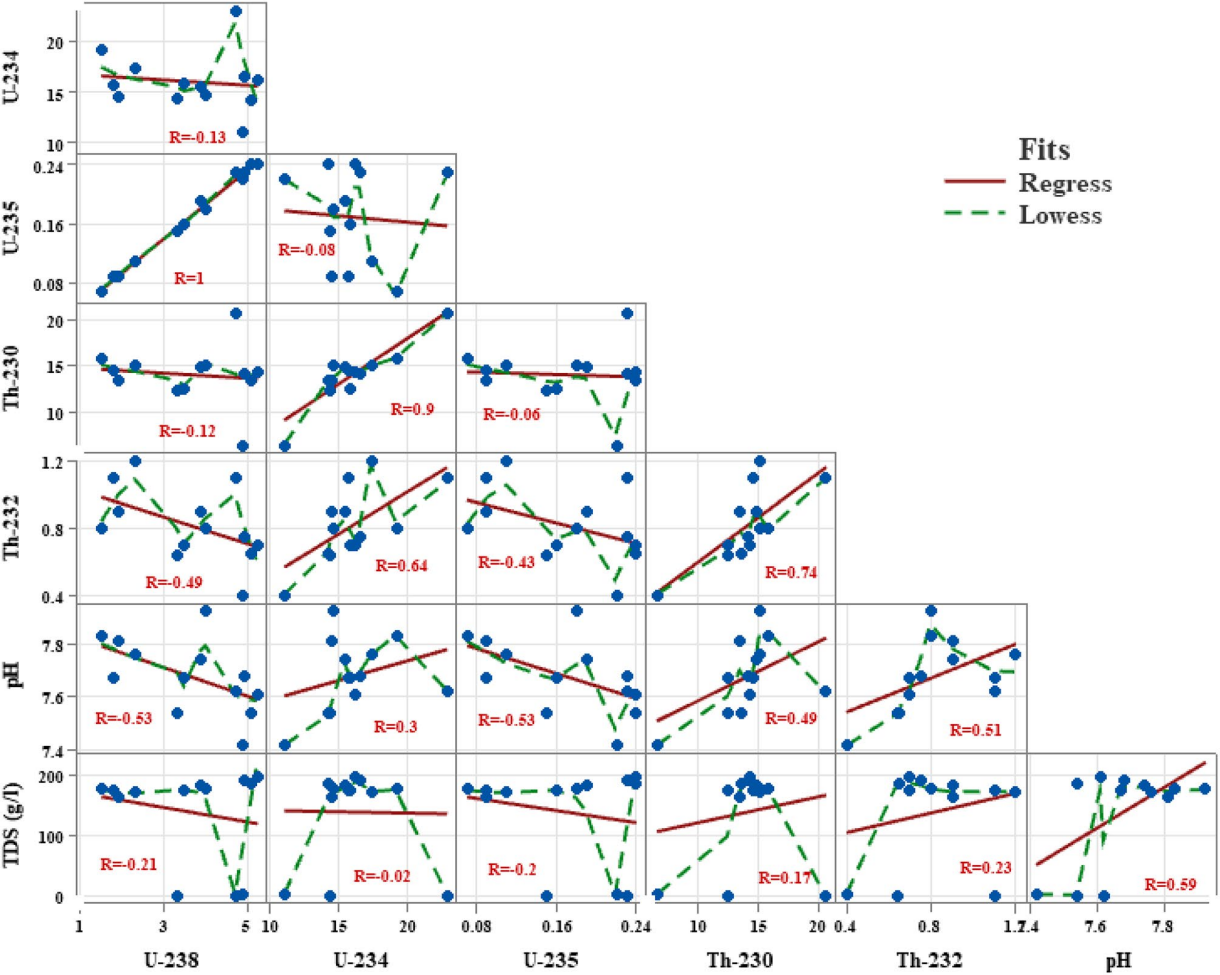
Correlation matrix between radionuclides isotopes (^238^U,^234^U,^235^U,^230^Th and ^232^Th) and environmental parameters (pH and TDS).

Correlation between environmental variables (pH and TDS) and radionuclide isotopes (^238^U, ^234^U, ^235^U, ^230^Th, and ^232^Th) are represented in (Fig. 3). According to the correlation, U-238 and U-235 had a weak negative connection with TDS and a moderately negative correlation with pH. This indicates that uranium increases with decreasing pH and TDS, but to different degrees. For this reason, the U content is higher in groundwater that has a more neutral pH, which is consistent with^46,47^. There is a weak positive correlation between Th-230, Th-232 and TDS, and a moderate positive correlation between Th-230, Th-232 and U-234 and pH. On the other hand, there is a strong positive correlation between Th-230, Th-232 and U-234 and a weak positive correlation between U-238 and U-234, which is contrary to expectations. According to these correlations, the pH value has a greater influence on the differentiation of the uranium concentration in water samples than the TDS value.

### Activity ratios

**Figure 4:**
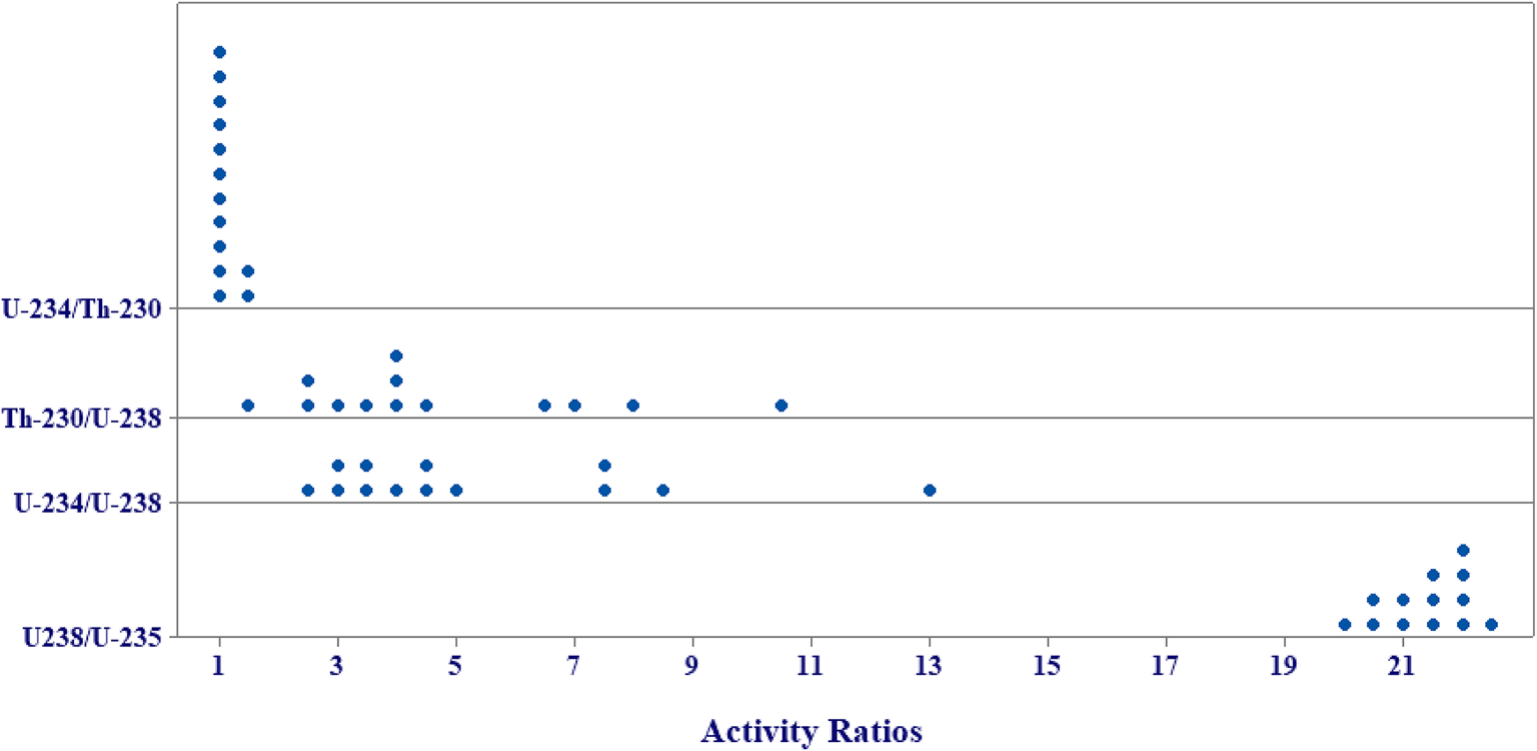
The activity ratios (ARs) of U-series in water samples of Siwa Oasis.

In closed geological systems, the activity ratios (ARs) ^234^U/^230^Th, ^230^Th/^238^U and ^234^U/^238^U appear to be equal to unity, but in these samples there are large radioactive disequilibria between U and its daughter (AR ≠ 1). For instance, the activity ratios (ARs) ^234^U/^230^Th, ^230^Th/^238^U and ^234^U/^238^U varied from 0.96 to 1.69, 1.33 to 10.60 and 2.25 to 12.82, respectively as shown in Table 4. From Fig. 4, ^234^U/^230^Th was very close to unity or hardly deviated from secular equilibrium in most of the samples except Spring (3), indicating disequilibrium. The equilibrium between ^234^U and ^230^Th indicates that Th-rich rocks in the aquifer have no discernible distribution pattern except that it is unexpectedly abundant in groundwater and this result is similar to the example analyzed by^41^.

**Table 4:** The activity ratios (ARs) of Uranium and its daughter nuclides in water samples.

Sample no	^234^U/^230^Th	^230^Th /^238^U	^234^U /^238^U	^238^U/^235^U
Zietoun Lake (1)	1.13 ± 0.53	2.73 ± 1.02	3.09 ± 1.16	21.88 ± 6.37
Zietoun Lake (2)	1.05 ± 0.49	2.66 ± 1.00	2.79 ± 1.05	21.25 ± 6.26
Zietoun Lake (3)	1.17 ± 0.55	2.87 ± 1.08	3.34 ± 1.26	21.52 ± 6.31
Aghormi Lake (1)	0.96 ± 0.45	3.78 ± 1.44	3.64 ± 1.40	22.33 ± 6.44
Aghormi Lake (2)	1.04 ± 0.49	3.83 ± 1.48	3.97 ± 1.53	20.47 ± 6.11
Aghormi Lake (3)	1.27 ± 0.60	3.57 ± 1.39	4.54 ± 1.77	21.88 ± 6.36
Siwa Lake (1)	1.07 ± 0.51	7.11 ± 2.99	7.63 ± 3.22	21.11 ± 9.79
Siwa Lake (2)	1.15 ± 0.54	6.57 ± 2.69	7.57 ± 3.10	20.91 ± 10.74
Siwa Lake (3)	1.08 ± 0.50	8.06 ± 3.42	8.72 ± 3.65	20.00 ± 9.49
Siwa Lake (4)	1.21 ± 0.57	10.60 ± 4.64	12.82 ± 5.61	21.43 ± 8.5
Spring (1)	1.11 ± 0.52	4.36 ± 1.65	4.84 ± 1.83	20.61 ± 6.14
Spring (2)	1.15 ± 0.54	3.75 ± 1.47	4.33 ± 1.70	22.13 ± 7.61
Spring (3)	1.69 ± 0.80	1.33 ± 0.50	2.25 ± 0.85	22.23 ± 6.42

As mentioned earlier, differential leaching of ^234^U from the host matrix leads to variations in the activity ratio (^234^U/^238^U) in the aquifer^48^. The ^234^U/^238^U ratios are explained either by oxidising waters close to recharge zones, which have high ^238^U contents and low ^234^U/^238^U activity ratios, or by reducing conditions, which have lower solubilities of ^238^U and higher ^234^U/^238^U activity ratios.Our results argue for reducing conditions, and a-recoil processes preferentially inject ^234^U into solution^38^. According to^49–51^, the average values of activity ratios (^234^U/^238^U) of groundwater ranged from 0.51 to 9.02. The ^234^U/^238^U ratio may occasionally move steadily to 5–20 in groundwater at mid- and high-latitudes of the Earth, and may even increase to 50 under certain circumstances^52–54^. In other words, the behaviour of uranium concentration in groundwater depends on the relationship between many variables, including pH, oxidation potential, CO_2_ partial pressure, the rock matrix of the aquifer and the flow of the groundwater stream. However, the highest activity ratios of uranium represent the accumulation of ^234^U due to the increasing interaction between water and rock over time^55^.

In the same context, the daughter nuclide (^234^U) passes most actively from surface coatings (carbonates, iron oxides and clay minerals) into the water, increasing the activity ratio of uranium isotopes in water^56^. The results suggest that the uranium series disequilibrium may be an important tool for tracking the migration of uranium series radionuclides in groundwater from different aquifers^48^. The ^238^U/^235^U ratio (AR) showed no relevant variation among the samples and approached the value of the natural uranium ratio of 21.7, as shown in Fig. 4.

**Figure 5:**
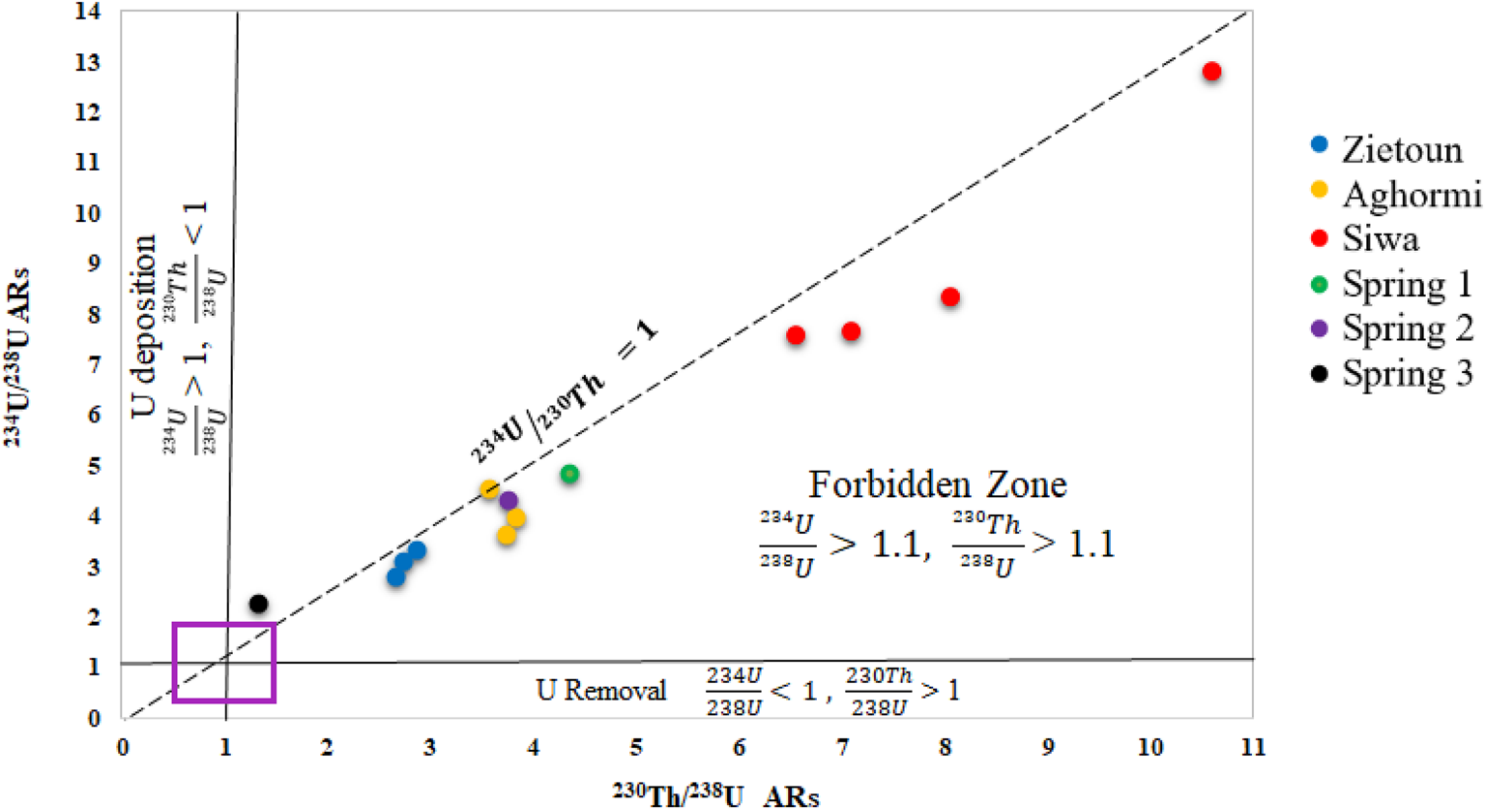
Thiel diagram of activity ratios of U-series in water samples of Siwa Oasis.

The Thiel diagram of ^230^Th/^238^U versus ^234^U/^238^U (Fig. 5) was used to identify the deposition or leaching (removal) of U at the sites; we rely here on Thiel's conceivable hypotheses^57^. According to this diagram, the forbidden zone contains all samples. It could be seen that the systems have difficulties that complicate uranium mobility in the forbidden zone, a complex geochemical zone. Datasets located in the forbidden zone may be caused by ongoing and conflicting uranium mobilisation activities^58^. The data set located in the forbidden zone could explain the relative rates of U gains and losses and the degree of U fractionation^59^.

### Uranium-series dating

The ages of groundwater determined in this study are shown in Table 5. An important source of uncertainty in U/Th dating is the correction of thorium not grown in by radioactive decay; isochron methods are effective mechanisms for reducing these uncertainties. Almost all samples have the activity ratio ^230^Th/^234^U =  < 1. Under these circumstances, the daughter's activity is in secular equilibrium with that of her mother. The activity ratios ^230^Th/^234^U <  = 1, interpreted as expressing fractures that are sealed or have lower transmissivity so that they are essentially hydraulically isolated from the main groundwater flow channel, indicate U accumulation^38^.

**Table 5:** Activity concentrations of ^230^Th*, isotopic activity ratios and ages (corrected and uncorrected) of uranium deposition in groundwater samples.

Sample no	Activity concentration (Bq/l)	Activity ratios					Age^a^ (Ka)	Age^b^ (Ka)
	^230^Th*	^230^Th/^234^U	^238^U/^234^U	^234^U/^232^Th	^230^Th/^232^Th	^230^Th*/^234^U		
Zietoun Lake (1)	11.61 ± 4.62	0.88 ± 0.42	0.32 ± 0.12	23.20 ± 11.81	20.20 ± 10.43	0.72 ± 0.37	163.62	114.42
Zietoun Lake (2)	11.01 ± 4.36	0.95 ± 0.46	0.35 ± 0.13	21.87 ± 11.31	20.86 ± 10.80	0.78 ± 0.40	193.01	130.99
Zietoun Lake (3)	11.26 ± 4.56	0.85 ± 0.46	0.29 ± 0.11	22.06 ± 11.01	18.93 ± 9.02	0.69 ± 0.35	153.60	105.73
Aghormi Lake (1)	11.87 ± 4.82	1.02 ± 0.42	0.27 ± 0.10	18.31 ± 9.041	18.75 ± 9.38	0.87 ± 0.42	217.62	138.15
Aghormi Lake (2)	11.37 ± 4.77	0.96 ± 0.47	0.25 ± 0.09	17.16 ± 8.261	16.54 ± 7.13	0.75 ± 0.39	189.01	117.83
Aghormi Lake (3)	9.763 ± 4.00	0.78 ± 0.30	0.22 ± 0.08	22.70 ± 11.59	17.85 ± 9.77	0.62 ± 0.32	129.71	89.84
Siwa Lake (1)	9.981 ± 4.31	0.93 ± 0.41	0.13 ± 0.05	16.11 ± 7.769	15.00 ± 7.19	0.63 ± 0.37	169.27	104.18
Siwa Lake (2)	10.40 ± 4.81	0.86 ± 0.41	0.13 ± 0.05	14. ± 6.591	12.58 ± 5.54	0.53 ± 0.34	149.41	86.68
Siwa Lake (3)	10.19 ± 4.62	0.96 ± 0.42	0.12 ± 0.05	13.63 ± 6.301	13.18 ± 6.61	0.62 ± 0.38	180.79	102.02
Siwa Lake (4)	12.77 ± 5.12	0.82 ± 0.32	0.07 ± 0.03	24.03 ± 11.81	19.87 ± 9.07	0.67 ± 0.34	135.97	98.12
Spring (1)	16.36 ± 6.67	0.90 ± 0.47	0.20 ± 0.07	20.83 ± 9.641	18.79 ± 8.83	0.70 ± 0.37	163.47	111.48
Spring (2)	9.947 ± 3.99	0.86 ± 0.49	0.23 ± 0.09	22.45 ± 11.69	19.45 ± 10.49	0.63 ± 0.36	153.38	106.93
Spring (3)	4.936 ± 2.05	0.59 ± 0.25	0.44 ± 0.16	27.52 ± 16.14	16.25 ± 9.59	0.44 ± 0.23	87.87	61.01

^a^Uncorrected age dating for samples.

^b^Corrected age dating for samples.

The ^230^Th/^232^Th activity ratios ranged from 12.58 ± 5.54 to 20.86 ± 10.80. According to^60^, if the ^230^Th/^232^Th activity ratio is less than 20, significant detrital contamination is assumed; the samples in this study, with the exception of Zietoun (1) and (2), were less than 20. The samples with ratios greater than 20 can be considered pure carbonate samples and correction may not be necessary. Even though our samples were corrected to determine the real age of the samples.

**Figure 6:**
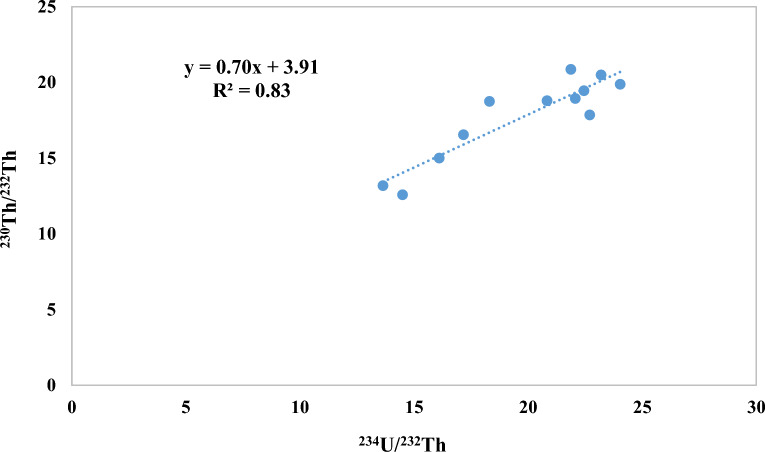
^230^Th/^232^Th vs ^234^U/^232^Th activity ratio isochron diagram.

**Figure 7:**
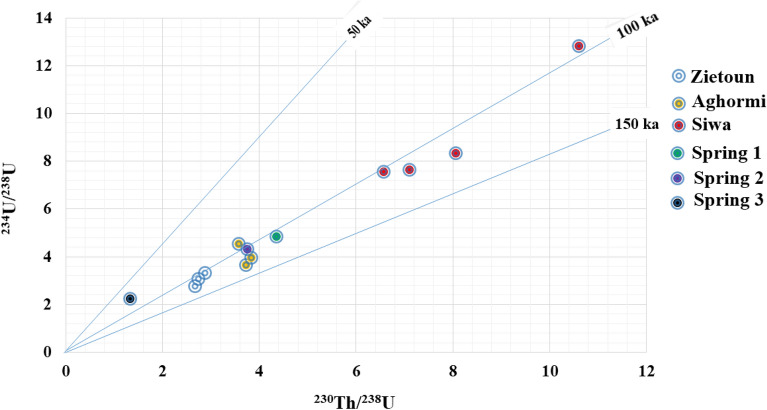
Cross plot of ^234^U/^238^U vs. ^230^Th/^238^U in water samples of Siwa Oasis.

The slope of the straight mixing line (isochron) in the diagram of ^230^Th/^232^Th AR versus ^234^U/^232^Th (AR) corresponds to the actual ^230^Th*/^234^U (AR), which is the crucial parameter for calculating ^230^Th*/U ages. The intersection of the isochron with the y-axis gives the actual [^230^Th/^232^Th] AR or the thorium index f^61^, as shown in Fig. 6. The isochron-corrected age of the samples corrected with a ^230^Th/^232^Th ratio was 3.91. The actual radiogenic ^230^Th and the age of the investigated samples were determined using the Eq. (2). The activity concentrations of Th*(radiogenic), ^230^Th/^234^U, ^238^U/^234^U, ^234^U/^232^Th, ^230^Th/^232^Th and ^230^Th*/^234^U were used to calculate the uncorrected ages and the correct ages as shown in Table 3. Plots of the ^234^U/^238^U activity ratios versus the detritus-corrected ^230^Th/^238^U activity ratios are also used to verify the closed system conditions (Fig. 7). The uncorrected ages of the uranium deposits in the water samples studied ranged from 87.87 to 193.01 Ka and the corrected ages ranged from 61.01 to 130.99 Ka, which is lower than the values found in the groundwater of Dakhla Oasis in the Western Desert of Egypt^2^ and consistent with the groundwater in the natural reference site Palmottu in Finland^37^.

## Conclusions

U-Th dating has found wide application in the fields of geology, environment and archaeology and has revolutionised Quaternary research. The U-series methodology depends on the efficient separation of uranium and thorium in environmental processes. The aim of our study was to use the long-lived radionuclides ^238^U-^234^U-^230^Th to assess the age of the groundwater (residence times) of the Siwa Oasis. The activity ratios (ARs) of the uranium series are ^234^U/^238^U > 1 and ^230^Th/^238^U > 1 in all samples and rather high ^234^U and ^230^Th concentrations in the groundwater. Our results indicated that the disequilibrium in the U series. In addition, the diagram of ^230^Th/^238^U and ^234^U/^238^U showed that all samples are in the forbidden zone where there was complicated U migration.

The samples are characterized by a moderate U content but an unusually high Th content. This could be the result of leaching of Th-bearing minerals in the deep aquifer. The thorium in the groundwater could be present as a colloidal component and not in true solution. In the same context, the activity ratio of ^238^U/^235^U (AR) deviates only slightly from the environmental value (21.7). The calculated ages of the U-series method approach varied between 87.87 and 193.01 Ka for the uncorrected age and between 61.01 and 130.99 Ka for the corrected age.

## Data Availability

The datasets and materials used during the current study are available from the corresponding author on reasonable request. All data generated or analysed during this study are included in this published article.
